# Dataset showing steel cold rolling process parameters for a 6-high cold rolling mill in Nigeria

**DOI:** 10.1016/j.dib.2018.03.081

**Published:** 2018-03-22

**Authors:** Emmanuel O. Ayuba, Christian.A. Bolu, Temitope M. John, Abiodun A. Abioye

**Affiliations:** a Department of Mechanical Engineering, Covenant University, Ota, Nigeria; b Department of Electrical and Information Engineering, Covenant University, Ota, Nigeria

**Keywords:** X-ray gauge, Accuracy, Automatic gauge control, Cold rolling mill, Steel rolling mill

## Abstract

The data contained in this article was acquired from the automatic gauge control system for a steel cold rolling mill production line in Nigeria. Accuracy is the one of the most important indices of productivity during a milling process. A total of 486 data points were obtained from selected feedback sensors located on the rolling mill machine via the control panel Human Machine Interface (HMI). The selected rolling parameters were gathered at different time intervals for different sample coils strips during the different milling stages. The data shows parameters such as actual thickness measured, x-ray gauge temperature, mill speed at both entry and exit and the mill power. This dataset could be used to analyze and improve the accuracy of the Automatic gauge control system and reduction in error in thickness variation.

**Specifications Table**

**Table:** 

Subject area	*Manufacturing Engineering*
More specific subject area	*Automatic Gauge Control system, Cold Steel Rolling Mills, X-ray gauge.*
Type of data	*Tables*
How data was acquired	*The rolling process parameters were gathered from the 6 high rolling mill of the steel cold rolling mill Line 1 via the Intelligent Mechatronics system (IMS) and Supervisory Control and Data (SCADA) program for the six milling passes to achieve the target thickness as final output.*
Data format	*Raw, filtered, analyzed, etc.*
Experimental factors	*N/A*
Experimental features	*The 486 data points were gathered from the rolling milling automatic gauge control system for a period of 400* *h at different time intervals. The data were gathered for the six milling passes to determine the error and deviation in target exit thickness measurements as the X-ray temperature, mill speed and mill power varies.*
Data source location	*A Steel Rolling Mill located in Abeokuta, Ogun State Nigeria*
Data accessibility	*Data are available within this article in the supplementary material section.*

**Value of the Data**

• The dataset for the thickness measurements can be analyzed to determine the efficiency of the rolling mill automatic gauge control system.
• The dataset can be used to investigate the direct effect of change in temperature of the X-ray gauge on the thickness variation at both sides of the mill.
• The dataset can be used to determine the accuracy level of the cold rolling mill production line.
• The dataset could be used to predict the most significance rolling mill process parameters.

## 1 Data

The following parameters were selected from the 6-high cold rolling mill of a steel cold rolling plant; located in Abeokuta, Ogun State, Nigeria. For each pass (thickness reduction stages in the milling process. Table 1 shows the description of the sensor (input) parameters retrieved from the HMI panel. Table 2, Table 3, Table 4, Table 5, Table 6, Table 7, Table 8, Table 9, Table 10 show the first order descriptive statistics for the input parameters.

**Table 1:** Description of iInput parameters.

***s/n***	***Parameter Code***	***Parameters***	***Description***
1	TTEn (mm)	Target entry thickness (mm)	This is the thickness measurement preset by the operator at the entry side of the mill for each pass before the mill process starts; the mill is set to carry milling process for passes [1].
2	TTEx (mm)	Target Exit Thickness (mm)	This is the desired exit thickness for each pass, this measurement determines the thickness of the strip sheet after every pass
3	ATEn (mm)	Actual Entry Thickness (mm)	This is the measurement derived from the X-ray sensing device positioned the entry point of the mill. This is the actual thickness of the sheet passing through the rolls.
4	ATEx (mm)	Actual Exit Thickness (mm)	This is the thickness of the strip after a milling process as taken place. The measurement is taken by the X-ray sensing device positioned at the exit side of the mill [2].
5	XTEx (°C)	Exit X-ray Temperature (°C)	This is the temperature in degree Celsius of the cooling chamber unit connected to the exit side of the X-ray source.
6	XTEn (°C)	Entry X-ray Temperature (°C)	This is the temperature in degree Celsius of the cooling chamber unit connected to the entry side of the X-ray source.
7	MP (kW)	Mill Power (kW)	This is the power in Kilowatts (kW) exerted on the backup rolls that cause a deformation in the strip sheet to be milled.
8	MSEn (mpm)	Entry Mill Speed (mpm)	The speed of drive of the coiler called the Payup reel at the entry side of the mill, the mill speed sustains tension across the mill. Its unit of measurement is in metres per minutes (mpm).
9	MSEx (mpm)	Exit Mill Speed (mpm)	The speed of the drive of the coiler called the Payoff reel at the exit side of the mill, this speed ensures that tension is maintained across the mill and ensures that the sheet are coiled up properly after the milling is completed. Its unit of measurement is in metres per minutes (mpm).

**Table 2:** Descriptive information for target thickness at entry (mm).

**Coil no.**	**PASS**	**Mean**	**Median**	**Mode**	**Maximum**	**Minimum**	**Standard deviation**	**Variance**	**Count**
1	1	1.8	1.8	1.8	1.8	1.8	0	0	2
	2	1.26	1.26	1.26	1.26	1.26	0	0	2
	3	0.81	0.81	0.81	0.81	0.81	0	0	6
	4	0.52	0.52	0.52	0.52	0.52	0	0	4
	5	0.33	0.33	0.33	0.33	0.33	0	0	6
	6	0.21	0.21	0.21	0.21	0.21	0	0	8
2	1	1.8	1.8	1.8	1.8	1.8	0	0	2
	2	1.26	1.26	1.26	1.26	1.26	0	0	5
	3	0.81	0.81	0.81	0.81	0.81	0	0	4
	4	0.52	0.52	0.52	0.52	0.52	0	0	6
	5	0.33	0.33	0.33	0.33	0.33	0	0	6
	6	0.21	0.21	0.21	0.21	0.21	0	0	9
3	1	1.8	1.8	1.8	1.8	1.8	0	0	8
	2	1.26	1.26	1.26	1.26	1.26	0	0	8
	3	0.81	0.81	0.81	0.81	0.81	0	0	15
	4	0.52	0.52	0.52	0.52	0.52	0	0	12
	5	0.33	0.33	0.33	0.33	0.33	0	0	11
	6	0.21	0.21	0.21	0.21	0.21	0	0	30
4	1	1.8	1.8	1.8	1.8	1.8	0	0	13
	2	1.26	1.26	1.26	1.26	1.26	0	0	9
	3	0.81	0.81	0.81	0.81	0.81	0	0	10
	4	0.52	0.52	0.52	0.52	0.52	0	0	14
	5	0.33	0.33	0.33	0.33	0.33	0	0	11
	6	0.21	0.21	0.21	0.21	0.21	0	0	19
5	1	1.8	1.8	1.8	1.8	1.8	0	0	18
	2	1.26	1.26	1.26	1.26	1.26	0	0	6
	3	0.81	0.81	0.81	0.81	0.81	0	0	6
	4	0.52	0.52	0.52	0.52	0.52	0	0	10
	5	0.33	0.33	0.33	0.33	0.33	0	0	16
	6	0.21	0.21	0.21	0.21	0.21	0	0	20
6	1	1.8	1.8	1.8	1.8	1.8	0	0	11
	2	1.26	1.26	1.26	1.26	1.26	0	0	12
	3	0.81	0.81	0.81	0.81	0.81	0	0	14
	4	0.52	0.52	0.52	0.52	0.52	0	0	19
	5	0.33	0.33	0.33	0.33	0.33	0	0	16
	6	0.21	0.21	0.21	0.21	0.21	0	0	19

**Table 3:** Descriptive Information for target thickness at exit (mm).

**Coil no.**	**PASS**	**Mean**	**Median**	**Mode**	**Maximum**	**Minimum**	**Standard deviation**	**Variance**	**Count**
1	1	1.26	1.26	1.26	1.26	1.26	0	0	2
	2	0.81	0.81	0.81	0.81	0.81	0	0	2
	3	0.52	0.52	0.52	0.52	0.52	0	0	6
	4	0.33	0.33	0.33	0.33	0.33	0	0	4
	5	0.21	0.21	0.21	0.21	0.21	0	0	6
	6	0.14	0.14	0.14	0.14	0.14	0	0	8
2	1	1.26	1.26	1.26	1.26	1.26	0	0	2
	2	0.81	0.81	0.81	0.81	0.81	0	0	5
	3	0.52	0.52	0.52	0.52	0.52	0	0	4
	4	0.33	0.33	0.33	0.33	0.33	0	0	6
	5	0.21	0.21	0.21	0.21	0.21	0	0	6
	6	0.14	0.14	0.14	0.14	0.14	0	0	9
3	1	1.26	1.26	1.26	1.26	1.26	0	0	8
	2	0.81	0.81	0.81	0.81	0.81	0	0	8
	3	0.52	0.52	0.52	0.52	0.52	0	0	15
	4	0.33	0.33	0.33	0.33	0.33	0	0	12
	5	0.21	0.21	0.21	0.21	0.21	0	0	11
	6	0.14	0.14	0.14	0.14	0.14	0	0	30
4	1	1.26	1.26	1.26	1.26	1.26	0	0	13
	2	0.81	0.81	0.81	0.81	0.81	0	0	9
	3	0.52	0.52	0.52	0.52	0.52	0	0	10
	4	0.33	0.33	0.33	0.33	0.33	0	0	14
	5	0.21	0.21	0.21	0.21	0.21	0	0	11
	6	0.14	0.14	0.14	0.14	0.14	0	0	19
5	1	1.26	1.26	1.26	1.26	1.26	0	0	18
	2	0.81	0.81	0.81	0.81	0.81	0	0	6
	3	0.52	0.52	0.52	0.52	0.52	0	0	6
	4	0.33	0.33	0.33	0.33	0.33	0	0	10
	5	0.21	0.21	0.21	0.21	0.21	0	0	16
	6	0.14	0.14	0.14	0.14	0.14	0	0	20
6	1	1.26	1.26	1.26	1.26	1.26	0	0	11
	2	0.81	0.81	0.81	0.81	0.81	0	0	12
	3	0.52	0.52	0.52	0.52	0.52	0	0	14
	4	0.33	0.33	0.33	0.33	0.33	0	0	19
	5	0.21	0.21	0.21	0.21	0.21	0	0	16
	6	0.14	0.14	0.14	0.14	0.14	0	0	19

**Table 4:** Descriptive information for actual thickness at entry (mm).

**Coil _no.**	**PASS**	**Mean**	**Median**	**Mode**	**Maximum**	**Minimum**	**Standard deviation**	**Variance**	**Count**
1	1.00	1.80	1.80	1.79	1.81	1.79	0.02	0.00	2
	2.00	1.24	1.24	1.23	1.26	1.23	0.03	0.00	2
	3.00	0.80	0.80	0.80	0.82	0.80	0.01	0.00	6
	4.00	0.52	0.51	0.51	0.53	0.51	0.01	0.00	4
	5.00	0.33	0.33	0.32	0.34	0.32	0.01	0.00	6
	6.00	0.21	0.21	0.21	0.21	0.21	0.00	0.00	8
2	1.00	1.78	1.78	1.77	1.78	1.77	0.00	0.00	2
	2.00	1.27	1.27	1.27	1.28	1.27	0.00	0.00	5
	3.00	0.80	0.80	0.79	0.81	0.79	0.01	0.00	4
	4.00	0.52	0.52	0.50	0.57	0.50	0.03	0.00	6
	5.00	0.33	0.33	0.33	0.34	0.33	0.00	0.00	6
	6.00	0.21	0.21	0.21	0.22	0.21	0.00	0.00	9
3	1.00	1.78	1.78	1.78	1.79	1.78	0.01	0.00	8
	2.00	1.26	1.26	1.25	1.28	1.24	0.01	0.00	8
	3.00	0.81	0.81	0.80	0.83	0.79	0.01	0.00	15
	4.00	0.52	0.52	0.51	0.53	0.50	0.01	0.00	12
	5.00	0.33	0.33	0.32	0.34	0.32	0.01	0.00	11
	6.00	0.21	0.21	0.21	0.22	0.21	0.00	0.00	30
4	1.00	1.79	1.78	1.78	1.81	1.77	0.01	0.00	13
	2.00	1.26	1.26	1.25	1.28	1.25	0.01	0.00	9
	3.00	0.81	0.81	0.80	0.83	0.79	0.01	0.00	10
	4.00	0.52	0.51	0.51	0.53	0.51	0.01	0.00	14
	5.00	0.33	0.34	0.34	0.34	0.33	0.00	0.00	11
	6.00	0.21	0.21	0.21	0.22	0.21	0.00	0.00	19
5	1.00	1.78	1.79	1.79	1.80	1.75	0.02	0.00	18
	2.00	1.27	1.27	1.27	1.27	1.26	0.01	0.00	6
	3.00	0.83	0.82	0.79	0.90	0.79	0.04	0.00	6
	4.00	0.51	0.52	0.51	0.53	0.49	0.01	0.00	10
	5.00	0.33	0.33	0.33	0.34	0.32	0.01	0.00	16
	6.00	0.21	0.21	0.21	0.22	0.20	0.00	0.00	20
6	1.00	1.79	1.79	1.79	1.80	1.78	0.01	0.00	11
	2.00	1.25	1.25	1.22	1.28	1.22	0.02	0.00	12
	3.00	0.81	0.81	0.80	0.82	0.79	0.01	0.00	14
	4.00	0.51	0.51	0.51	0.53	0.50	0.01	0.00	19
	5.00	0.33	0.33	0.33	0.34	0.32	0.01	0.00	16
	6.00	0.21	0.21	0.21	0.22	0.21	0.00	0.00	19

**Table 5:** Descriptive information for actual thickness at exit (mm).

**Coil_no.**	**PASS**	**Mean**	**Median**	**Mode**	**Maximum**	**Minimum**	**Standard deviation**	**Variance**	**Count**
1	1	1.27	1.27	1.26	1.27	1.26	0.01	0	2
	2	0.8	0.8	0.8	0.81	0.8	0.01	0	2
	3	0.52	0.52	0.51	0.53	0.51	0.01	0	6
	4	0.33	0.33	0.33	0.33	0.33	0	0	4
	5	0.21	0.21	0.21	0.22	0.21	0	0	6
	6	0.14	0.14	0.14	0.14	0.13	0	0	8
2	1	1.24	1.24	1.23	1.26	1.23	0.02	0	2
	2	0.81	0.82	0.8	0.82	0.8	0.01	0	5
	3	0.53	0.52	0.52	0.54	0.52	0.01	0	4
	4	0.33	0.33	0.32	0.35	0.32	0.01	0	6
	5	0.21	0.21	0.21	0.21	0.21	0	0	6
	6	0.14	0.15	0.13	0.16	0.13	0.01	0	9
3	1	1.26	1.26	1.25	1.28	1.24	0.01	0	8
	2	1.00	0.81	0.79	0.79	0.79	0.01	0	8
	3	0.51	0.51	0.5	0.53	0.5	0.01	0	15
	4	0.33	0.33	0.33	0.34	0.32	0.01	0	12
	5	0.21	0.21	0.21	0.22	0.21	0	0	11
	6	0.13	0.14	0.14	0.15	0.12	0	0	30
4	1	1.26	1.26	1.27	1.28	1.24	0.01	0	13
	2	0.8	0.8	0.8	0.81	0.79	0	0	9
	3	0.51	0.52	0.52	0.52	0.5	0.01	0	10
	4	0.33	0.33	0.33	0.34	0.33	0	0	14
	5	0.19	0.21	0.21	0.22	0.03	0.06	0	11
	6	0.14	0.14	0.13	0.16	0.13	0.01	0	19
5	1	1.26	1.26	1.24	1.28	1.24	0.02	0	18
	2	0.81	0.81	0.79	0.82	0.79	0.01	0	6
	3	0.53	0.53	0.51	0.55	0.51	0.01	0	6
	4	0.34	0.33	0.33	0.4	0.32	0.02	0	10
	5	0.21	0.21	0.22	0.22	0.21	0	0	16
	6	0.14	0.14	0.13	0.16	0.13	0.01	0	20
6	1	1.27	1.27	1.27	1.28	1.25	0.01	0	11
	2	0.81	0.81	0.8	0.84	0.79	0.01	0	12
	3	0.51	0.51	0.51	0.53	0.51	0.01	0	14
	4	0.33	0.33	0.33	0.38	0.31	0.01	0	19
	5	0.21	0.21	0.21	0.22	0.21	0	0	16
	6	0.13	0.13	0.13	0.14	0.13	0	0	19

**Table 6:** Descriptive information for X-ray Temp at entry (°C).

**Coil_no.**	**PASS**	**Mean**	**Median**	**Mode**	**Maximum**	**Minimum**	**Standard deviation**	**Variance**	**Count**
1	1	30.6	30.6	30.6	30.6	30.6	0	0	2
	2	32.1	32.1	32	32.2	32	0.14	0.02	2
	3	32.08	32.4	32.4	32.6	30.3	0.88	0.77	6
	4	31.38	31.25	31	32	31	0.48	0.23	4
	5	32.52	32.55	32.6	32.7	32.3	0.15	0.02	6
	6	31.8	31.8	30.9	32.7	30.9	0.96	0.93	8
2	1	31.3	31.3	31	31.6	31	0.42	0.18	2
	2	33.14	33.1	33	33.3	33	0.15	0.02	5
	3	31.65	31.3	31.3	33	31	0.91	0.83	4
	4	31.98	31.65	31.6	32.7	31.6	0.56	0.31	6
	5	32.43	32.45	32.5	32.7	32.2	0.18	0.03	6
	6	32.82	33	32	33.6	32	0.69	0.47	9
3	1	30.95	31.1	31.1	31.4	30.2	0.41	0.17	8
	2	31.23	31.6	31.6	31.6	30.6	0.52	0.27	8
	3	30.92	31.7	31.8	32	28.9	1.27	1.6	15
	4	31.5	31.6	31.6	31.7	30.9	0.26	0.07	12
	5	54.97	29.5	28.8	30.8	28.8	0.83	0.07	11
	6	30.76	30.8	31	31.5	30.2	0.36	0.13	30
4	1	30.82	31.2	29.1	31.9	29.1	1.01	1.02	13
	2	31.6	31.6	31.6	31.6	31.6	0	0	9
	3	29.95	29.4	28.8	31.6	28.8	1.19	1.43	10
	4	31.57	31.6	31.6	31.6	31.3	0.08	0.01	14
	5	30.17	30	30.2	32.1	28.9	1.07	1.15	11
	6	30.65	30.5	31.6	31.6	29.9	0.7	0.5	19
5	1	30.84	31.05	31.9	31.9	28.9	1.17	1.37	18
	2	30.83	30.85	30.9	30.9	30.7	0.08	0.01	6
	3	30.67	31.05	31.9	32	28.9	1.46	2.12	6
	4	31.04	31	31	31.4	30.8	0.16	0.02	10
	5	29.95	29.6	28.9	31.3	28.7	1	1	16
	6	30.91	31	31	31.2	30.4	0.26	0.07	20
6	1	31.43	31.6	31.6	31.9	30.4	0.57	0.33	11
	2	29.76	29.5	29.5	31.2	28.7	0.96	0.92	12
	3	31.14	31.5	31.9	32.1	29	1.08	1.16	14
	4	31.48	31.5	31.5	31.5	31.2	0.07	0.01	19
	5	31.18	30.9	30.9	32.2	30.2	0.64	0.41	16
	6	31.53	31.6	31.6	31.6	31	0.18	0.03	19

**Table 7:** Descriptive information for X-ray Temp at exit (°C).

**Coil_no.**	**PASS**	**Mean**	**Median**	**Mode**	**Maximum**	**Minimum**	**Standard deviation**	**Variance**	**Count**
1	1	31	31	31	31	31	0	0	2
	2	31.25	31.25	30.4	32.1	30.4	1.2	1.45	2
	3	31.85	31.7	31.6	32.7	31	0.61	0.38	6
	4	32.45	32.45	32.4	32.5	32.4	0.06	0	4
	5	32	32	32	32	32	0	0	6
	6	30.85	30.8	30.6	31.5	30.5	0.34	0.11	8
2	1	31.25	31.25	31	31.5	31	0.35	0.13	2
	2	31.66	31	31	32.7	31	0.9	0.82	5
	3	31.75	32	32	32	31	0.5	0.25	4
	4	32.18	32.15	32	32.5	31.9	0.25	0.06	6
	5	32.88	33.1	33.1	33.1	31.8	0.53	0.28	6
	6	31.69	30.8	30.7	33.1	30.5	1.22	1.5	9
3	1	31.13	31	31	32	31	0.35	0.13	8
	2	29.84	29.55	29.1	31.3	28.9	0.94	0.88	8
	3	31.36	31.6	31.6	31.7	31	0.31	0.09	15
	4	30.01	29.6	29	31.8	29	1.05	1.1	12
	5	30.09	29.9	29.9	31	29.8	0.45	0.2	11
	6	30.91	31.05	30.6	32.1	28.9	0.85	0.72	30
4	1	31.01	31	31.2	31.2	30.4	0.27	0.07	13
	2	29.66	29.5	30.5	30.6	28.8	0.74	0.54	9
	3	31	31	31	31	31	0	0	10
	4	50.64	30.25	28.9	30.9	28.9	0.74	0.55	14
	5	31.26	31.1	31	31.8	30.7	0.42	0.18	11
	6	30.79	30.8	30.8	32.1	28.9	1.09	1.18	19
5	1	30.15	29.9	29.9	31.6	29.2	0.77	0.59	18
	2	29.65	29.45	28.9	31.1	28.9	0.8	0.65	6
	3	30.82	30.65	30.6	31.7	30.6	0.44	0.19	6
	4	29.83	29.5	27.8	31.7	27.8	1.31	1.71	10
	5	30.91	31.4	29.9	31.7	29.8	0.83	0.68	16
	6	31.02	30.95	30.1	32.1	29.7	0.86	0.74	20
6	1	30.56	30.6	30.6	30.6	30.4	0.07	0	11
	2	29.25	28.9	28.9	30.6	28.7	0.57	0.33	12
	3	30.54	30.6	30.6	30.7	30	0.17	0.03	14
	4	30.31	30.6	30.6	31.9	28.8	0.82	0.68	19
	5	30.59	30.6	30.6	30.8	30.4	0.09	0.01	16
	6	30.32	30.4	29.2	32	28.9	0.97	0.94	19

**Table 8:** Descriptive information for mill speed at entry in (mpm).

**Coil_no.**	**PASS**	**Mean**	**Median**	**Mode**	**Maximum**	**Minimum**	**Standard deviation**	**Variance**	**Count**
1	1	98	98	31	165	31	94.75	8978	2
	2	183.5	183.5	124	243	124	84.15	7080.5	2
	3	326	325.5	322	331	322	3.16	10	6
	4	597.25	597.5	594	600	594	2.5	6.25	4
	5	606	606	607	610	603	2.53	6.4	6
	6	597.5	612	610	617	490	43.51	1892.86	8
2	1	218.5	218.5	217	220	217	2.12	4.5	2
	2	253.76	227	219	356.8	219	58.16	3382.89	5
	3	473.65	500.05	329	565.5	329	101.27	10255	4
	4	540.68	622.65	358	643.2	358	141.11	19910.66	6
	5	390.17	390	390	395	384	3.76	14.17	6
	6	322.89	275	237	471	237	91.27	8330.11	9
3	1	170.81	184.05	184.1	184.2	145.4	18.56	344.64	8
	2	223.5	224	225	231	217	4.34	18.86	8
	3	265.47	312	315	315	34	90.73	8231.7	15
	4	340	377.5	130	386	130	75.26	5663.45	12
	5	384.55	408	411	413	278	47.17	2225.47	11
	6	353.42	393	289	431	29.7	91.34	8342.74	30
4	1	185.02	212	199.8	253.6	30.9	81.88	6703.64	13
	2	228.11	228	228	233	224	2.67	7.11	9
	3	314.6	314.5	314	318	309	2.84	8.04	10
	4	353.29	384.5	390	391	282	44.2	1954.07	14
	5	381.82	400	400	410	318	34.91	1218.96	11
	6	338.26	374	374	401	200	68.55	4699.76	19
5	1	69.75	71	48	122.1	33.9	21.36	456.07	18
	2	195	197.5	198	200	185	5.59	31.2	6
	3	265.83	309.5	124	319	124	78.85	6217.37	6
	4	315.6	322.5	191	386	191	69.06	4769.16	10
	5	304.75	345.5	350	368	194	62.55	3912.6	16
	6	280.45	268	235	356	230	46.6	2171.1	20
6	1	256.35	256.4	256	256.8	256	0.31	0.09	11
	2	199.92	218.5	84	315	84	60.97	3716.99	12
	3	305.79	312.5	312	324	222	24.97	623.41	14
	4	356.58	384	382	398	226	52.94	2802.26	19
	5	277.63	283.5	331	334	196	54.48	2967.98	16
	6	325.26	338	391	396	246	50.37	2537.32	19

**Table 9:** Descriptive information for mill speed at exit in (mpm).

**Coil_no.**	**PASS**	**Mean**	**Median**	**Mode**	**Maximum**	**Minimum**	**Standard deviation**	**Variance**	**Count**
1	1	98.95	98.95	30.9	167	30.9	96.24	9261.61	2
	2	270.5	270.5	169	372	169	143.54	20604.5	2
	3	509	509	506	512	506	2.37	5.6	6
	4	381.25	381	379	384	379	2.22	4.92	4
	5	389.67	390.5	382	394	382	4.13	17.07	6
	6	379.38	388.5	390	393	316	25.82	666.55	8
2	1	337.5	337.5	333	342	333	6.36	40.5	2
	2	335.58	357.2	251.7	360	251.7	47.05	2214.01	5
	3	515.93	500.2	498.4	564.9	498.4	32.68	1067.7	4
	4	611.68	633.2	633.2	643.7	558	41.04	1684.63	6
	5	603	603	603	605	601	1.41	2	6
	6	470.89	379	368	669	368	143.43	20572.86	9
3	1	170.15	174.5	183	183	145.2	14.81	219.27	8
	2	350.75	351	351	353	349	1.39	1.93	8
	3	402.33	488	489	493	60	142.67	20355.38	15
	4	530.59	593.5	596	600.1	190	126.13	15909.16	12
	5	598.09	634	431	643	431	73.44	5392.89	11
	6	568.9	618	460	666	325	109.85	12066.16	30
4	1	188.05	199	187	254	30.7	78.93	6230.72	13
	2	360.33	360	359	363	358	1.58	2.5	9
	3	491.8	490.5	490	497	488	3.12	9.73	10
	4	547.64	602.5	603	606	420	72.49	5254.71	14
	5	564.27	624	627	631	405	90.79	8242.22	11
	6	535.05	598	598	620	321	107.93	11649.72	19
5	1	115.44	102.5	86	207	55	45.5	2069.91	18
	2	302.83	301.5	301	310	299	3.87	14.97	6
	3	414.67	485.5	489	489	209	118.8	14113.07	6
	4	480.4	475	409	596	256	114.46	13101.82	10
	5	463.25	539	350	546	224	109.28	11942.87	16
	6	432.1	423	330	563	327	89.74	8052.62	20
6	1	256	257	257	258	253	1.55	2.4	11
	2	321.67	349.5	148	487	148	87.42	7641.88	12
	3	474.5	489.5	492	493	378	38.87	1511.19	14
	4	562.11	597	606	608	400	68.06	4631.65	19
	5	433.31	449	508	516	313	82.29	6771.3	16
	6	508.84	537	542	627	377	85.63	7332.14	19

**Table 10:** Descriptive information for mill power (kW).

**Coil_no.**	**PASS**	**Mean**	**Median**	**Mode**	**Maximum**	**Minimum**	**Standard deviation**	**Variance**	**Count**
1	1	1179	1179	1178	1180	1178	1.41	2	2
	2	1185	1185	1183	1187	1183	2.83	8	2
	3	1693	1711.5	1561	1760	1561	79.08	6253.47	6
	4	1557	1556.5	1554	1559	1554	2.08	4.33	4
	5	1217	1217.5	1207	1227	1207	6.65	44.17	6
	6	978	1000	1000	1015	803	71.29	5082.86	8
2	1	37.5	37.5	33	42	33	6.36	40.5	2
	2	1711	1667	1632	1804	1632	82.79	6854.8	5
	3	1611	1780	1102	1782	1102	339.34	115148.67	4
	4	1513	1515	1504	1521	1504	6.77	45.77	6
	5	1258	1257.5	1254	1263	1254	3.58	12.8	6
	6	1100	1100	1108	1108	1090	6.8	46.28	9
3	1	795.3	743.5	739	937	739	86.63	7505.36	8
	2	1698	1698	1689	1711	1689	7.4	54.7	8
	3	1381	1692	1692	1705	110	551.84	304525.11	15
	4	1428	1545.5	811	1563	811	273.19	74635.15	12
	5	1181	1268	1266	1275	703	182.31	33237.36	11
	6	921.9	1000.5	996	1077	542	172.67	29816.4	30
4	1	1113	1145	899	1198	899	100.1	10019.92	13
	2	1715	1718	1700	1725	1700	8.24	67.94	9
	3	1693	1694.5	1694	1700	1684	4.99	24.93	10
	4	1397	1555	1556	1576	1019	207.7	43138.26	14
	5	1217	1250	1250	1256	1016	73.43	5391.62	11
	6	855.9	952	969	1011	610	153.09	23436.1	19
5	1	522.1	405	310	1135	180	285.86	81713.4	18
	2	1480	1481.5	1463	1495	1463	14.68	215.37	6
	3	1448	1646	798	1772	798	393.93	155178.57	6
	4	1041	1123	1142	1175	572	185.6	34447.82	10
	5	909.9	1110	1110	1125	409	290.14	84181.93	16
	6	709.9	746	516	934	516	157.35	24760.34	20
6	1	1426	1415	1389	1480	1389	29.02	842.16	11
	2	1453	1676	222	1699	222	446.62	199471.72	12
	3	1676	1701.5	1701	1716	1434	75.94	5766.37	14
	4	1402	1518	943	1592	943	238.45	56860.06	19
	5	936.3	1037	1050	1106	648	160.7	25825.56	16
	6	855.4	900	621	1015	621	140.12	19633.25	19

The data gathered from the various parameters were analyzed for each phases or stages of the rolling mill process. A pass is the defined as a phase/ stage in which the material is reduced to a predetermined range of thickness [3]. Each coil was subjected to six passes; the target exit thickness for each pass is given below:

**First Pass**: First stage of the milling process and the material is reduced from its initial thickness of 1.800 mm to a target thickness of 1.2600 mm;
**Second Pass**: Second stage of the milling process where the entry thickness is 1.260 mm and the target exit thickness is 0.806 mm;
**Third Pass:** Third stage of the milling process, its entry thickness is 0.806 mm and target exit thickness is 0.516 mm;
**Fourth pass:** Fourth stage of the milling process, its entry thickness is 0.516 mm and target exit thickness measurement is 0.330 mm;
**Fifth Pass:** Fifth stage of the milling process with 0.330 mm and exit target thickness measurement is 0.211 mm;
**Sixth Pass:** Final stage of milling with entry thickness measurement of 0.211 mm and exit thickness measurement is 0.135 mm.

## 2 Experimental design, materials, and methods

This data were manually collected from the Supervisory Control and Data Acquisition (SCADA) via the operators Human Machine Interface (HMI).The Supervisory Control and Data Acquisition system (SCADA) provides a detailed real time monitoring of the numerous feedback parameters [4], [5] from installed sensors during the manufacturing process [6], [7]. An average of three readings was recorded for each pass with minimum time interval of 3 minutes for the various parameters [8]. The X-ray thickness gauge sensor specifications are presented in Table 11. The tolerance range for thickness measurement for the reversible cold rolling mill is ±0.001 μm [1]. The Analysis of Variance (ANOVA) for rolling parameters are given in Table 12, Table 13, Table 14, Table 15.

**Table 11:** X-ray gauge specifications.

Measurement range	3500–8000 μm
Source	160 KV
Operating values	85KV, 2 mA
Sensitivity	0.1%
Max high voltage	100 KV
Maximum tube current	10 mA
Maximum continuous output	1 kW
Resolution	0.001 μm
Mill Type & direction configuration	Cold, Reversible
Maximum cooling chamber temperature	35 °C
Cooling chamber flow rate	4litres/Min

**Table 12:** ANOVA analysis of rolling parameters mill speed at entry and exit.

		**Sum of squares**	**df**	**Mean square**	***F***	**Sig.**
Mill_Speed_entry_side_mpm	Between Groups	2252428.224	5	450485.645	52.539	0.000
	Within Groups	3266827.338	381	8574.350		
	Total	5519255.562	386			
Mill_Speed_exit_mpm	Between Groups	5472997.442	5	1094599.488	110.776	0.000
	Within Groups	3764746.031	381	9881.223		
	Total	9237743.473	386

**Table 13:** ANOVA analysis of parameters X-ray Temp at entry and exit.

		**Sum of squares**	**df**	**Mean square**	***F***	**Sig.**
Xray_Temp_at_entry	Between Groups	786.378	5	157.276	0.788	0.559
	Within Groups	76090.613	381	199.713		
	Total	76876.991	386			
Xray_Temp_at_exit	Between Groups	947.494	5	189.499	0.941	0.454
	Within Groups	76724.734	381	201.377		
	Total	77672.228	386

**Table 14:** ANOVA analysis of parameters actual thickness at entry and exit.

		**Sum of squares**	**df**	**Mean square**	***F***	**Sig.**
Actual_Thickness_at_entry_mm	Between Groups	113.717	5	22.743	203031.301	0.000
	Within Groups	0.043	381	0.000		
	Total	113.760	386			
Actual_Thickness_at_exit_mm	Between Groups	13975.476	5	2795.095	1.731	0.127
	Within Groups	615260.434	381	1614.857		
	Total	629235.911	386

**Table 15:** ANOVA analysis of parameters Mill power.

	**Sum of Squares**	**df**	**Mean Square**	***F***	**Sig.**
Between Groups	32150337.441	5	6430067.488	81.468	0.000
Within Groups	30071288.745	381	78927.267		
Total	62221626.186	386			
